# Multiple sporadic schwannomas in a previously radiated field

**DOI:** 10.1016/j.radcr.2024.09.051

**Published:** 2024-09-20

**Authors:** Jeffrey Rosenthal, Chukwuemeka Okoro, Naomi Walker, Scott Nelson, Nicholas Bernthal, Varand Ghazikhanian

**Affiliations:** a Department of Radiological Sciences, UCLA Health System, Los Angeles, CA, USA; b Department of Pathology and Laboratory Medicine, UCLA Health System, Los Angeles, CA, USA; c Department of Orthopaedic Surgery, UCLA Health System, Los Angeles, CA, USA

**Keywords:** Peripheral nerve sheath tumor, Radiation-induced schwannoma, CT-guided biopsy

## Abstract

Peripheral nerve sheath tumors are a heterogenous group of predominantly benign tumors of neurogenic origin that arise outside of the central nervous system and include schwannomas and neurofibromas. These tumors often occur sporadically, however multiple lesions are generally associated with genetic syndromes such as neurofibromatosis (type 1 and 2) and schwannomatosis, and occasionally these tumors and their malignant variations are associated with a history of radiation treatment. Multiple benign schwannomas in an irradiated field have seldom been reported in the literature. We describe a case of a 49-year-old male with a history of right sided irradiated testicular cancer who presented with 2 histologically confirmed benign schwannomas in the right pelvic wall and right psoas muscle.

## Introduction

Benign peripheral nerve sheath tumors (PNSTs) represent 10%-12% of benign soft tissue neoplasms [1]. The vast majority of PNSTs are sporadic, solitary neurofibromas or schwannomas, however genetic syndromes such as neurofibromatosis and schwannomatosis are well known to predispose to multiple PNSTs. While there are many individual and overlapping features, neurofibromatosis type 1 (NF-1) is categorically defined by multiple neurofibromas and characteristic skin abnormalities, neurofibromatosis type 2 (NF-2) by bilateral vestibular schwannomas, and schwannomatosis by multiple schwannomas [2].

Limited reports describe multiple sporadic PNSTs, and this case presents a patient with 2 sporadic extra-cranial histologically proven schwannomas. Given the patient's history of radiation treatment in the same region, we discuss the entity of radiation-induced schwannomas, an uncommon late complication of radiation therapy.

## Case report

A 49-year-old male with a past medical history of right sided testicular cancer treated with radiation and orchiectomy 22 years prior presented with a 5-month history of intermittent radiating right medial thigh pain. The pain spread to his lower back and was not associated with physical activity. He denied weakness, bowel or bladder changes, and recent trauma. His physical exam demonstrated full strength and an intact neurovascular examination on the right lower extremity. There was no tenderness to palpation. His skin examination was nonrevealing. Basic laboratory markers were unremarkable.

**Fig. 1:**
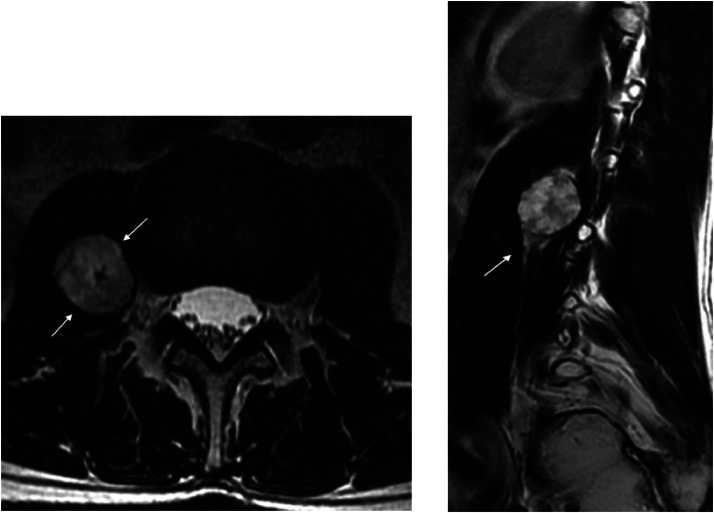
Axial T2- weighted image (A) demonstrates a well circumscribed hyperintense mass within the right psoas muscle (white arrows). Sagittal T1 -weighted (B) image shows the peripheral rim of T1 hyperintensity along the long axis of the lesion consistent with a split fat sign (arrow).

Initially the patient underwent MRI of the lumbar spine, which demonstrated a well circumscribed, hyperintense on T2-weighted sequence, intensely enhancing lesion within the right psoas muscle with a peripheral rim of T1 hyperintensity along the long axis of the lesion consistent with a split fat sign. The paraspinal location suggested that the lesion likely arose from ventral right L2 nerve root (Fig. 1).

**Fig. 2:**
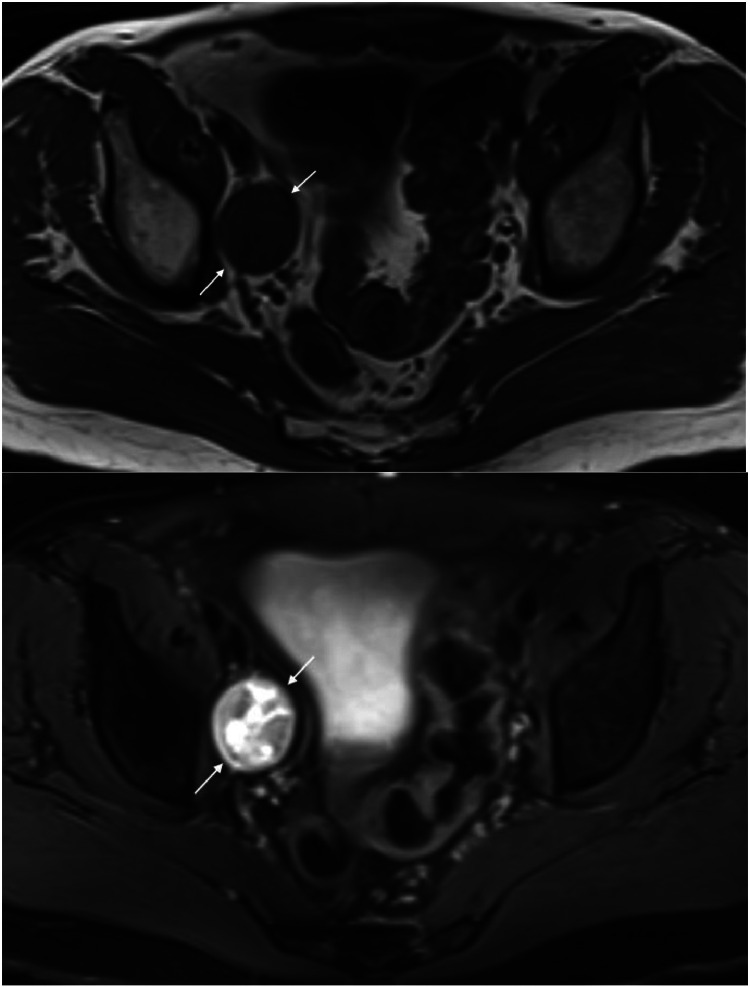
Axial T1-weighted (A) and T2-weighted fat suppressed (B) images demonstrate an ovoid well circumscribed 6.4 × 3.3 × 3.1 cm right pelvic mass with intermediate to hypointense T1 signal, and heterogeneous T2 signal (white arrows).

Given the fact that his symptoms of medial thigh and groin pain did not anatomically correlate with an L2 lesion, additional imaging of the pelvis was performed. The patient underwent a dedicated MRI of the pelvis, which demonstrated an additional ovoid, well circumscribed right pelvic mass with intermediate to hypointense signal on T1-weighted sequence, heterogeneous signal on T2-weighted sequence, and heterogenous diffusion restriction. There was no peritumoral edema (Fig. 2).

**Fig. 3:**
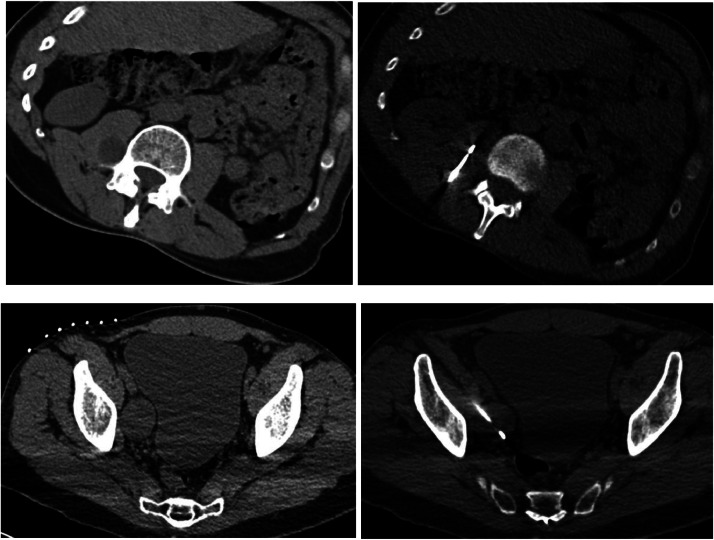
Axial CT images demonstrate the right psoas (A) and pelvic masses (C) and subsequent CT guided needle placement for biopsies of these lesions; right psoas mass (B) and pelvic mass (D).

A PET-CT demonstrated FDG avidity of both the right pelvic wall mass, with an SUV of 3.9, and right psoas muscle mass, with an SUV of 4.7. Given the size of the masses as well as the intermediate uptake of the masses on PET, in conjunction with the patient's oncologic history, we elected to proceed with biopsies. The patient subsequently underwent CT-guided right L2-3 and L4-L5 nerve blocks and core needle biopsies of the masses under moderate sedation (Fig. 3).

**Fig. 4:**
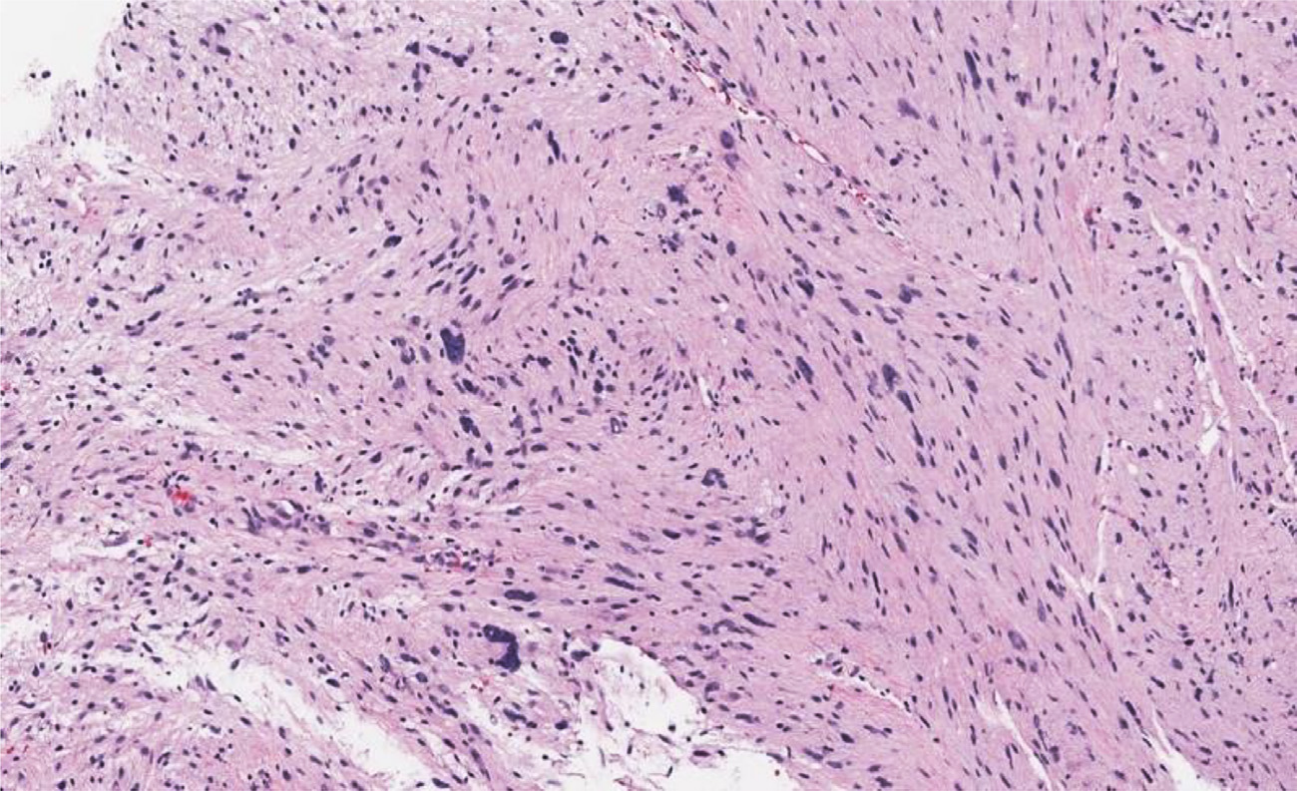
Sections demonstrated a spindle cell neoplasm arranged in fascicles. The spindle cells contained variable hyperchromatic nuclei, some with small nucleoli. While most nuclei were bland, scattered cells demonstrated enlarged, bizarre, hyperchromatic features. Mitotic activity was not increased. The background had a fine neuropil appearance. Immunohistochemistry showed the cells to be strongly and diffusely positive for S100 and SOX10, and negative for caldesmon. The histological and immunohistochemical features were consistent with schwannoma with so-called ancient change.

The pathology of the right psoas muscle mass was consistent with a schwannoma and similarly, the right pelvic wall mass pathology demonstrated a schwannoma with ancient change (Fig. 4). After discussing treatment options including surgery, the patient and oncologic surgeon chose to follow the benign schwannomas with surveillance imaging.

## Discussion

Benign PNSTs include neurofibromas and schwannomas, and may be found in multiple as a component of well-described genetic syndromic conditions, such as neurofibromatosis and schwannomatosis. Sporadic schwannomas are more often found in men than women, within 20-50 years of age [3]. Histologically, schwannomas are typically well-circumscribed, demonstrate variably cellular Antoni A and Antoni B regions, nuclear palisading, and notably stain strongly and diffusely positive for S-100 [1]. Schwannomas are usually found in the head, thorax, and abdomen, and occur in the pelvis, as in our patient, 0.3%-3.2 % of the time [4].

MRI is the preferred imaging modality for detecting and characterizing PNSTs. Schwannomas are generally spherical or ovoid in shape with a relatively smooth border. They are hypointense or isointense to skeletal muscle on T1-weighted sequences and hyperintense on T2-weighted sequences, often with central nonenhancing fluid signal and peripheral rim enhancement. About 66% have been shown to undergo cystic change [5].

Retroperitoneal schwannomas are most often found in the paravertebral regions, and when intramuscular, demonstrate characteristic MRI signs. The most common MRI sign is the split fat sign, best seen on T1-weighted images, demonstrated by prominent fatty tissue surrounding the lesion [6]. Another sign is the low signal margin, which signifies a hypointense rim on T2-weighted images representing the epineurium. Lastly, the fascicular sign, on T2-weighted and gadolinium enhanced images, is defined by a hyperintense rim with ring-like structures centrally. More common for neurofibromas is the target sign, which is low signal centrally with a peripheral ring of high signal on T2-weighted images [7]. It is difficult to differentiate sporadic versus syndromic PNSTs on MRI, however multiple lesions along a nerve are the most useful sign that predicts an underlying syndrome. Additionally, sporadic lesions have been suggested to demonstrate more internal heterogeneity [8].

One of the lesions in this case was histologically categorized as an ancient schwannoma, which refers to degeneration with nuclear atypia and diffuse hypocellular regions. On MRI this may correlate to T2 hemorrhagic, cystic, or necrotic change [9].

The vast majority of sporadic PNSTs are schwannomas over neurofibromas, and it is uncommon for a patient to have multiple sporadic lesions as in this case. Our case is one of few reports of multiple pathology proven schwannomas in a patient without a genetic syndrome [10]. Additionally, sporadic schwannomas rarely arise within the psoas muscle [11].

While clinical history and imaging features may suggest benignity, biopsy is often required to rule out malignancy or entertain alternative diagnoses. Despite anecdotal and historical literature suggesting otherwise, CT-guided core needle biopsy of PNSTs is safe and accurate, with a low complication rate limited to exacerbation of pain [12].

Our case is particularly unique given the patient's remote history of testicular cancer (22 years prior to presentation) treated with radiation. The medical chart at the time of the patient's radiation treatment was not available, thus the exact radiation dose could not be obtained, however the general irradiation field for right sided testicular cancer would suggest that the tumors arose within the same region. While preradiation CT images were not available, review of previous radiology reports prior to the patient's presentation do not demonstrate masses in the right psoas muscle and right pelvis.

Radiation induced benign PNSTs are rare and described in case series and individual case reports. These tumors must meet defined criteria in order to be deemed radiation-induced, known as “Cahan's criteria” which includes a location within the field of radiotherapy, at least a 3-year latency period, and an absence of predisposing genetic conditions. Most research, however limited, has demonstrated an association between radiation and malignant PNSTs [13]. However, radiation induced benign PNSTs have seldom been described, both sporadic and in patients with neurofibromatosis [14]. On average, radiation induced malignant PNSTs have been shown to develop approximately 14 years after radiation exposure, while interestingly, as seen in this case, radiation induced benign PNSTs develop on average 25 years after exposure [13,15]. It has been postulated that low doses of radiation, less than 10 Gy, may induce both benign and malignant tumors, while high doses, over 30 Gy, induce malignant tumors. The overwhelming majority of reported radiation induced schwannomas are within the spine or eighth cranial nerve distribution, and very few are outside of the central nervous system [15].

In conclusion, this case is one of few reported cases of multiple pathology proven benign schwannomas in a patient without a genetic syndromic condition. It raises awareness regarding the development of benign PNSTs as a rare late complication of irradiation.

## Patient consent

Verbal informed consent for release of all published information, including medical information and imaging, was obtained from the patient described in this report. In order to maintain patient privacy, the patient information was de-identified.

Upon manuscript submission, the patient was not able to be reached for written consent despite multiple efforts.
